# Appraisal of the causal effect of plasma caffeine on adiposity, type 2 diabetes, and cardiovascular disease: two sample mendelian randomisation study

**DOI:** 10.1136/bmjmed-2022-000335

**Published:** 2023-01-31

**Authors:** Susanna C Larsson, Benjamin Woolf, Dipender Gill

**Affiliations:** 1 Unit of Cardiovascular and Nutritional Epidemiology, Institute of Environmental Medicine, Karolinska Institutet, Stockholm, Sweden; 2 Department of Surgical Sciences, Uppsala University, Uppsala, Sweden; 3 School of Psychological Science, University of Bristol, Bristol, UK; 4 Faculty of Epidemiology and Population Health, London School of Hygiene and Tropical Medicine, London, UK; 5 Medical Research Council Integrative Epidemiology Unit, University of Bristol, Bristol, UK; 6 Department of Epidemiology and Biostatistics, School of Public Health, Imperial College London, London, UK; 7 Chief Scientific Advisor Office, Research and Early Development, Novo Nordisk, Copenhagen, Denmark

**Keywords:** Cardiology, Diabetes mellitus, Epidemiology, Genetics, medical, Preventive medicine, Public health

## Abstract

**Objective:**

To investigate the potential causal effects of long term plasma caffeine concentrations on adiposity, type 2 diabetes, and major cardiovascular diseases.

**Design:**

Two sample mendelian randomisation study.

**Setting:**

Genome-wide association study summary data for associations of two single nucleotide polymorphisms associated with plasma caffeine at the genome-wide significance threshold (rs2472297 near the *CYP1A2* gene and rs4410790 near the *AHR* gene) and their association with the outcomes.

**Participants:**

Primarily individuals of European ancestry participating in cohorts contributing to genome-wide association study consortia.

**Main outcome measures:**

Outcomes studied were body mass index, whole body fat mass, whole body fat-free mass, type 2 diabetes, ischaemic heart disease, atrial fibrillation, heart failure, and stroke.

**Results:**

Higher genetically predicted plasma caffeine concentrations were associated with lower body mass index (beta −0.08 standard deviation (SD) (95% confidence interval −0.10 to −0.06), where 1 SD equals about 4.8 kg/m^2^ in body mass index, for every standard deviation increase in plasma caffeine) and whole body fat mass (beta −0.06 SD (−0.08 to −0.04), 1 SD equals about 9.5 kg; P<0.001) but not fat-free mass (beta −0.01 SD (−0.02 to −0.00), 1 SD equals about 11.5 kg; P=0.17). Higher genetically predicted plasma caffeine concentrations were associated with a lower risk of type 2 diabetes in two consortia (FinnGen and DIAMANTE), with a combined odds ratio of 0.81 ((95% confidence interval 0.74 to 0.89); P<0.001). Approximately half (43%; 95% confidence interval 30% to 61%) of the effect of caffeine on type 2 diabetes was estimated to be mediated through body mass index reduction. No strong associations were reported between genetically predicted plasma caffeine concentrations and a risk of any of the studied cardiovascular diseases.

**Conclusions:**

Higher plasma caffeine concentrations might reduce adiposity and risk of type 2 diabetes. Further clinical study is warranted to investigate the translational potential of these findings towards reducing the burden of metabolic disease.

**WHAT IS ALREADY KNOWN ON THIS TOPIC**

**WHAT THIS STUDY ADDS**

- Genetically predicted, lifelong, higher plasma caffeine concentrations were associated with lower body mass index and fat mass, as well as a lower risk of type 2 diabetes
- Approximately half of the effect of caffeine on type 2 diabetes was estimated to be mediated through body mass index reduction

**HOW MIGHT THIS STUDY AFFECT RESEARCH, PRACTICE, OR POLICY**

- Long term clinical studies investigating the effect of caffeine intake on fat mass and type 2 diabetes risk are warranted

## Introduction

 Caffeine (1,3,7-trimethylxanthine) is a widely consumed psychoactive substance. The main sources of caffeine globally are from coffee, tea, and soda drinks.^1^,^3^ Considering the extensive intake of caffeine worldwide,^1^,^3^ even its small metabolic effects could have important health implications.

Caffeine has thermogenic effects^4^,^9^ and has been implicated in reducing weight, body mass index (BMI), and fat mass in short term randomised controlled trials.^10^,^12^ Hence, a high caffeine intake might lower the risk of diseases related to adiposity, such as type 2 diabetes and cardiovascular disease. Evidence from observational studies supports an inverse association between coffee consumption and risk of type 2 diabetes, with all identified studies reporting a statistically significant or non-significant inverse association.^13^ In a dose-response meta-analysis, the risk of type 2 diabetes decreased by 7% for each cup per day increase of caffeinated coffee (an average cup contains around 70–150 mg caffeine) and by 6% for each cup per day increase of decaffeinated coffee.^13^ Observational findings on the association between coffee consumption and risk of ischaemic heart disease,^3 14^ stroke,^14 15^ and cardiovascular disease mortality^16^,^18^ are less consistent with both inverse and positive associations reported by different studies. However, observational studies cannot reliably infer causality because coffee consumption is associated with other factors (eg, other dietary and lifestyle factors, ethnic group, and socioeconomic status),^19^ potentially resulting in confounded associations. Furthermore, the specific effect of caffeine on the risk of cardiometabolic diseases might be difficult to disentangle from the other compounds included in caffeinated drinks and foods.^13^ Randomised controlled trials on caffeine consumption and chronic diseases are costly and difficult to implement. To our knowledge, no randomised controlled trials have been published on the effect of caffeine on risk of developing type 2 diabetes and cardiovascular disease. Nevertheless, a randomised controlled trial of the effect of coffee consumption on insulin sensitivity and other biological risk factors for type 2 diabetes in 126 adults who were overweight and had no sensitivity to insulin showed that consumption of four cups per day of caffeinated coffee for 24 weeks had no effect on insulin sensitivity or biological mediators of insulin resistance. This study did show an association with a reduction in fat mass and urinary creatinine concentration.^12^

The metabolism of caffeine occurs largely in the liver by the cytochrome P450 isoform 1A2 (CYP1A2).^20^ Genetic variations near genes *CYP1A2* and *AHR* (which regulates the expression of *CYP1A2*) are associated with plasma concentrations of caffeine.^20^ Individuals who carry genetic variants that are associated with slower caffeine metabolism consume, on average, less coffee but have higher plasma caffeine concentrations.^20^ This finding probably relates to individuals with a slow metabolism of caffeine consequently consuming less coffee and caffeine than people who have a fast caffeine metabolism, to reach or retain the concentrations of caffeine required for the desired psychostimulant effects.^20^

We leveraged genetic variants in caffeine metabolism through the mendelian randomisation framework to investigate the causal effects of long term exposure to higher plasma caffeine concentrations on adiposity, type 2 diabetes, and major cardiovascular diseases. The purpose of mendelian randomisation analysis is to improve causal inference by use of genetic variants that are reliably and strongly associated with the exposure as unbiased proxy indicators. As genetic variants are fixed at conception, individuals with genetic variants that are associated with higher plasma caffeine concentrations will, on average, be exposed to higher caffeine concentrations throughout their life compared with people with variants associated with lower plasma caffeine.

## Methods

### Study design and assumptions

**Figure 1:**
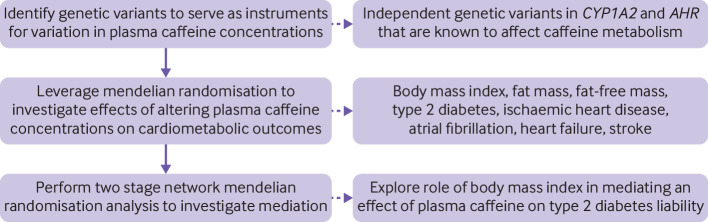
Schematic overview of the study design

We used a two sample mendelian randomisation design with summary genetic data obtained from genome-wide association studies. An overview of the study design is shown in figure 1. The genetic variants that we used as instrumental variables in mendelian randomisation analysis were expected to fulfill the three assumptions: strongly associated with the exposure (relevance assumption); not associated with potential confounders (independence assumption); and affect the outcome via the exposure and not via any other pathway (exclusion restriction assumption).

### Data sources

Single nucleotide polymorphisms (SNPs) that are associated with plasma caffeine were obtained from a genome-wide association meta-analysis of 9876 individuals of European ancestry from six population based studies.^20^ That study identified genome-wide significant associations of SNPs near *CYP1A2* and *AHR* loci with plasma caffeine concentrations.^20^ The SNPs within each locus were in linkage disequilibrium (*r*^2^ ranging from 0.21 to 0.96 in European populations), therefore, we selected the strongest signal at each locus, that is, rs2472297 at *CYP1A2* (P=1.0×10-20) and rs4410790 at *AHR* (P=1.8×10-13), as instrumental variables in the mendelian randomisation analyses. Estimates of the associations of the caffeine SNPs were obtained for BMI (whole body fat mass) from the GIANT consortium^21^ and for body composition (whole body fat-free mass) from the Medical Research Council's Integrative Epidemiology Unit. These estimates were extracted from the MR-Base platform.^22^ The corresponding summary genetic data for type 2 diabetes and cardiovascular disease subtypes were obtained from the FinnGen (R7 data),^23^ DIAMANTE (European),^24^ CARDIoGRAMplusC4D (including UK Biobank data),^25^ HERMES,^26^ and MEGASTROKE (restricted to individuals of European ancestry)^27^ consortia. Summary genetic data for atrial fibrillation were obtained from a meta-analysis of six studies.^28^ Details of the used data sources are presented in the online supplemental table 1. All summary statistics used in the mendelian randomisation analyses are shown in the online supplemental table 2.

### Statistical analysis

The mendelian randomisation estimate for the association of plasma caffeine with each outcome was computed by dividing the estimate of the SNP and outcome association (beta coefficient or log odds ratio) by the estimate of the SNP and caffeine association (ie, Wald ratio). This calculation used a standard deviation (SD) unit, which was estimated from the standardised (z) scores representing the change in plasma caffeine per effect allele from the caffeine genome-wide association study.^20^ The mendelian randomisation estimates for the two caffeine associated SNPs were then combined according to the inverse-variance weighted method. We combined the results for two studies using a random-effects meta-analysis (DerSimonian and Laird method), and calculated heterogeneity between studies using Cochran’s *Q* test. Results were deemed statistically significant at the Bonferroni corrected threshold of P<0.006 (P=0.05, eight studied outcomes). An association with a P value between 0.006 and 0.05 was considered suggestive evidence of an association.

As a follow-up analysis, we further conducted a two step network mendelian randomisation mediation analysis to explore the extent to which any effect of caffeine on type 2 diabetes might be mediated through BMI.^29^ The proportion mediated was calculated as the estimated effect of caffeine consumption on BMI multiplied by the estimated effect of BMI on type 2 diabetes. When estimating the association of genetically predicted BMI with type 2 diabetes, we selected genome-wide significant SNPs from the GIANT genome-wide association study on BMI (n=483) to be instrumental variables, as described previously. For the type 2 diabetes genetic association estimates, we performed a random-effects meta-analysis of FinnGen and DIAMANTE. In addition to the inverse variance weighted meta-analysis of the Wald ratios specific to the SNPs, we implemented three estimators that were deemed to be robust for pleiotropy (ie, when a genetic variant associates with more than one phenotype) as a sensitivity analysis: MR-Egger, weighted median, and weighted mode.^30^ The standard error of the mediated effect was then estimated by use of bootstrap simulations with 100 000 iterations.^31^

Most widely employedstatistical sensitivity analyses that use mendelian randomisation methods to detect and adjust for pleiotropy cannot be conducted with only two instrumental variables. To assess potential pleiotropy of the SNPs associated with caffeine, we instead performed a phenome-wide association analysis in the MR-Base platform (lookup on 1 August 2022).^22^ Stata and R software were used to analyse the data and to create the forest plot figure.

### Patient and public involvement

We did not engage patients or members of the public when designing this study, interpreting the results, or drafting the manuscript. They were also not involved in the dissemination plans of this study and we have no plans to disseminate the results to research participants and public communities.

## Results

**Figure 2:**
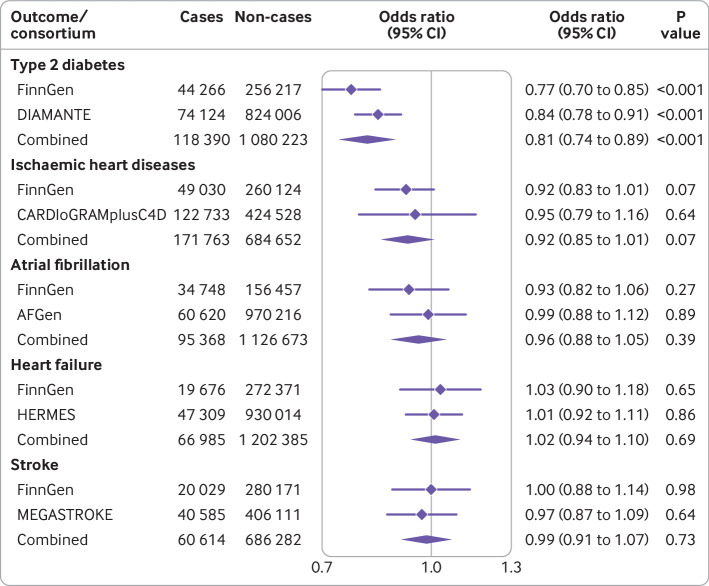
Genetically predicted higher plasma caffeine concentration and risk of type 2 diabetes and major cardiovascular diseases. The odds ratios with their corresponding 95% confidence intervals (CIs) are scaled per one standard deviation increase in plasma caffeine concentration. Cochran’s *Q* test statistic for heterogeneity between study specific estimates: *Q*=2.39 (P=0.12) for type 2 diabetes, *Q*=0.13 (P=0.72) for ischaemic heart disease, *Q*=0.48 (P=0.49) for atrial fibrillation, *Q*=0.08 (P=0.78) for heart failure, and *Q*=0.11 (P=0.74) for stroke

Characteristics of the studies used for the two sample mendelian randomisation analyses are shown in online supplemental table 1. All but one study encompassed participants of European ancestries only.^25^ Genetically predicted higher plasma concentrations of caffeine were associated with lower BMI (beta −0.08 SD (95% confidence interval −0.10 to −0.06), where 1 SD equals about 4.8 kg/m^2^, for every SD increase in plasma caffeine; P<0.001) and whole body fat mass (beta −0.06 SD (−0.08 to −0.04), 1 SD equals about 9.5 kg; P<0.001) but had a non-significant association with fat-free mass (beta −0.01 SD (−0.02 to −0.00), 1 SD equals about 11.5 kg; P=0.17). Additionally, genetically predicted higher plasma caffeine concentrations were associated with a lower risk of type 2 diabetes in both the FinnGen (odds ratio 0.77 (95% confidence interval 0.70 to 0.85) per SD increase in plasma caffeine; P<0.001) and the DIAMANTE consortia (0.84 (0.78 to 0.91); P<0.001); the combined odds ratio of type 2 diabetes per SD increase in plasma caffeine was 0.81 ((0.74 to 0.89); P<0.001) with no evidence of heterogeneity between the two studies (*Q*=2.39, P=0.12) (figure 2). Both SNPs were strongly associated with lower BMI, fat mass, and type 2 diabetes risk (all P<0.005) (online supplemental table 2). No strong association was observed between genetically predicted plasma caffeine with risk of ischaemic heart disease, atrial fibrillation, heart failure, or stroke, and no heterogeneity was noted among studies (*Q*<0.48, P>0.49 (figure 2)).

**Figure 3:**
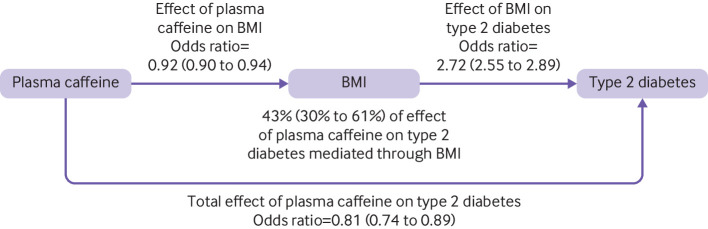
Causal directed acyclic graph showing the total effect of plasma caffeine on type 2 diabetes risk and the effect mediated by body mass index. The presented mendelian randomisation effect estimates with their corresponding 95% confidence intervals (shown in parenthesis) are scaled per one standard deviation increase in plasma caffeine concentration. BMI=body mass index

When performing mediation analysis to investigate the proportion of the effect of caffeine on type 2 diabetes liability that was mediated via BMI reduction, we detected heterogeneity in the inverse-variance weighted mendelian randomisation estimate (P<0.001, see online supplemental table 3 for full results). We, therefore, used the weighted median estimator for the mediation analysis because this estimator is both reasonably efficient and robust to pleiotropy. Our two step mediation analysis reported that the odds of type 2 diabetes per SD increase in plasma caffeine related to mediation through BMI was 0.91 (95% confidence interval 0.90 to 0.94). This finding suggested that 43% (30% to 61%) of the protective effect of plasma caffeine on type 2 diabetes was mediated through BMI (figure 3).

In the phenome-wide association analysis performed in the MR-Base platform, 39 763 phenotype associations were available for the *CYP1A*2 SNP and 40 553 were available for *AHR*. The alleles that increase plasma caffeine of the two SNPs were, in addition to lower coffee and tea consumption and lower BMI, associated with higher water and cereal intake and with renal biomarkers, including creatinine, urea, albumin, phosphate, sodium, and cystatin C at the genome-wide significance threshold (online supplemental tables 4 and 5). The SNP in the *CYP1A2* locus was additionally associated with higher peak expiratory flow, platelet distribution width and volume, diastolic blood pressure, and impedance of arm and lower leg fat percentage. The SNP in the *AHR* locus was further associated with higher concentrations of potassium in urine and liver biomarkers bilirubin and alkaline phosphatase, improved lipid profile, lower glycated haemoglobin concentrations, and lower concentrations of the protein sex hormone binding globulin, which was adjusted for BMI.

## Discussion

### Principal findings

In this mendelian randomisation investigation aimed at deciphering the possible long term causal effects of increased plasma caffeine on cardiometabolic outcomes, genetically predicted higher plasma caffeine concentrations were associated with lower BMI and whole body fat mass. Furthermore, genetically predicted higher plasma caffeine concentrations were associated with a lower risk of type 2 diabetes. Approximately half of the effect of caffeine on type 2 diabetes liability was estimated to be mediated through BMI reduction. No evidence suggested an association of genetically predicted higher plasma caffeine concentrations with ischaemic heart disease, atrial fibrillation, heart failure, and stroke.

### Comparison with other studies

The association between plasma caffeine concentrations and type 2 diabetes risk has been scarcely investigated. Nonetheless, the association of caffeinated or total coffee consumption with the risk of type 2 diabetes has been examined in many observational studies,^13^ which collectively support an inverse dose-response relation. Our mendelian randomisation finding suggests that caffeine might, at least in part, explain the inverse association between coffee consumption and risk of type 2 diabetes. In contrast to observational findings, no clear association has been reported between genetically predicted coffee consumption and type 2 diabetes in mendelian randomisation analyses.^3 32 33^ A one sample mendelian randomisation analysis based on the Copenhagen General Population Study showed no association of a allele score for coffee intake, consisting of SNPs near *CYP1A2* and *AHR* genes, with risk of type 2 diabetes.^32^ Two sample mendelian randomisation studies based on data from the Diabetes Genetics Replication And Meta‐analysis (DIAGRAM) consortium suggested a positive association between risk of type 2 diabetes and consumption of coffee (by 12 SNPs) and caffeine (from coffee, tea, or both; by 35 SNPs).^3 33^ Our findings for plasma caffeine might superficially seem inconsistent and contradictory to previous mendelian randomisation analyses of coffee and caffeine consumption. However, this discrepancy is expected because the genetic variants in the two genomic regions that are associated with higher plasma caffeine concentrations, due to potentially slower metabolism of caffeine, are also associated with lower coffee and caffeine consumption,^20^ which was also observed in our phenome-wide association analysis. In addition to different exposures (plasma caffeine concentrations *v* coffee or caffeine intake), the genetic method in our study was confined to SNPs located in genes encoding enzymes with an established role in caffeine metabolism, either directly via the CYP1A2 enzyme or indirectly (AHR) by the regulation of *CYP1A2* expression. Studies based on a larger set of instrumental variables^3 33^ included several SNPs in genes with a less clear or unknown role in predicting higher coffee and caffeine consumption,^34^ which can increase the risk of bias from pleiotropy.

Data for plasma caffeine and risk of cardiovascular disease are scarce, but several observational and mendelian randomisation studies have reported results on coffee consumption and cardiovascular disease incidence and mortality. Evidence from observational studies indicates that, compared with no coffee consumption, moderate consumption of coffee (three to five cups/day) is associated with lower risk of ischaemic heart disease^14 16^ and stroke.^14 15^ Observational studies further suggest that moderate coffee consumption might be associated with lower risk of heart failure^35 36^ but not with atrial fibrillation.^37^ Previous mendelian randomisation studies have not found any significant association between genetically predicted coffee consumption and total cardiovascular disease^38 39^ or specific cardiovascular diseases, including ischaemic heart disease and stroke.^3 38 40 41^ Given that the association between coffee consumption and cardiovascular disease risk seems to be non-linear, our mendelian randomisation study on plasma caffeine and previous mendelian randomisation studies on coffee consumption might have been unable to detect significant associations. Another possibility is that the observed associations in these observational studies are driven by residual confounding or reverse causation. Additionally, our mendelian randomisation analyses that investigated cardiovascular outcomes might have been under-powered due to low phenotypic variance explained by the used instrumental variables. Impaired glycaemic control increases cardiovascular risk, thereby suggesting that a protective effect of higher caffeine concentrations on diabetes liability would also reduce cardiovascular risk. The 95% confidence interval for the cardiovascular outcomes suggested that any possible protective effect of plasma caffeine concentrations on ischaemic heart disease is unlikely to be larger than 15% or more harmful than a 1% increase, likewise, for atrial fibrillation, a maximum of a 12% protective effect and 5% increase in harm.

As in previous mendelian randomisation studies,^33^ we found a strong association between genetically predicted BMI and type 2 diabetes risk. The magnitude of the association was somewhat stronger in our mendelian randomisation analysis than in previous mendelian randomisation studies,^33^ which might be related to different instruments used for BMI and different data sources for type 2 diabetes.

### Biological mechanisms

In terms of mechanisms, caffeine might reduce BMI and fat mass by increasing thermogenesis^4^,^9^ and fat oxidation.^42^,^44^ The effect of caffeine on energy expenditure is dose-dependent and the thermogenic response is positively correlated with the response in plasma caffeine.^9^ A daily intake of 100 mg of caffeine has been estimated to increase energy expenditure by approximately 100 kcal (418.4 kJ) per day,^8^ which could consequently lower the risk of developing obesity. The elevated energy expenditure attributable to caffeine consumption might be mediated through increased thermogenesis of brown adipose tissue.^45^ An alternative mechanism whereby higher caffeine concentrations reduce adiposity is through enhanced satiety^13^ and suppressed energy intake.^46^ The observed association between genetically predicted higher plasma caffeine concentrations with lower type 2 diabetes risk is partly mediated by reduced overall body fat, which is strongly causally associated with this disease.^33 47^ Our mendelian randomisation mediation analyses support this hypothesis.

The genetic alleles that are associated with higher plasma caffeine concentrations are also associated with lower coffee and tea consumption. Thus, an alternative mechanism is that individuals who carry those alleles have a lower risk of type 2 diabetes, compared with those with other sets of alleles,because of lower exposure to any other substances in coffee (eg, diterpenes) or tea that increase the risk of type 2 diabetes.^48^ In this regard, the plasma caffeine raising allele for the SNP in the *AHR* locus was associated with lower concentrations of triglycerides, low density lipoprotein cholesterol, and the protein sex hormone binding globulin, adjusted for BMI, as well as higher concentrations of bilirubin and alkaline phosphatase. The association with triglycerides and cholesterol might be related to lipid raising diterpenes (ie, cafestol and kahweol) present in unfiltered coffee.^48^ Coffee and caffeine consumption has been reported to be associated with sex hormone binding globulin^49^ as well as certain biomarkers of liver function and liver cancer.^50^

### Strengths and limitations of the study

An important strength of this study is the mendelian randomisation design, which minimises bias due to reverse causation because genetic variants are fixed at conception and cannot be changed by disease status. The study design also reduces potential effects of confounding factors because the genetic variants associated with the exposure under study are generally not associated with environmental exposures and other self-adopted behaviours, except for caffeine containing beverage consumption in this mendelian randomisation study. Another important strength is that the genetic instrument for plasma caffeine comprised SNPs in genes with a known role in caffeine metabolism, thereby reducing risk of bias due to pleiotropy. Additionally, both SNPs were strongly associated with type 2 diabetes risk in two independent large scale consortia, strengthening the evidence of causality. A limitation is that possible non-linear relations between plasma caffeine and the outcomes could not be explored within the current two sample mendelian randomisation design. Another shortcoming is that we could not conduct sensitivity analyses using commonly employed mendelian randomisation methods for detecting possible pleiotropy, such as MR-Egger regression and MR-PRESSO, because those approaches require at least three or more instrumental variables. Nevertheless, we conducted phenome-wide association analysis and found that only a few of the associated phenotypes (coffee and tea consumption and renal biomarkers) had a stronger association with the specific outcome phenotypes under study than plasma caffeine concentration did, despite the much larger sample sizes of these studies (about 7 to 80 times larger than in the plasma caffeine study). This finding implies that the associated phenotypes are probably downstream consequences of higher plasma caffeine, rather than biasing pleiotropic effects. Another limitation is that although our mendelian randomisation study provides evidence of a causal effect of plasma caffeine on reducing adiposity and type 2 diabetes risk, the extent of the association does not inform on the potential impact of any clinical or public health intervention. The use of only two SNPs for caffeine exposure reduced the power of the mendelian randomisation analyses. Additionally, because genetic predictors of plasma caffeine concentrations in people who are not of European ethnicity are not established and our data sources comprised European individuals only, our findings might not be generalisable to populations of non-European ethnicity.

### Conclusion

This mendelian randomisation study found evidence to support causal associations of higher plasma caffeine concentrations with lower adiposity and risk of type 2 diabetes. Randomised controlled trials are warranted to assess whether non-caloric caffeine containing beverages might play a role in reducing the risk of obesity and type 2 diabetes.

## Supplementary material

10.1136/bmjmed-2022-000335 online supplemental file 1

## Data Availability

All data relevant to the study are included in the article or uploaded as supplementary information.
