# Supplementary material for: Appraisal of the causal effect of plasma caffeine on adiposity, type 2 diabetes, and cardiovascular disease: two sample mendelian randomisation study
Source: BMJ Med. 2023 Jan 31;2(1):e000335. doi: 10.1136/bmjmed-2022-000335 (PMC9978685; doi:10.1136/bmjmed-2022-000335)
Supplement: Supplementary file 2 [file bmjmed-2022-000335.reviewer_comments.pdf]

PEER REVIEW HISTORY

BMJ Medicine publishes all reviews undertaken for accepted manuscripts. Reviewers are asked to complete a checklist review form and are provided with free text boxes to elaborate on their assessment. These free text comments are reproduced below.

ARTICLE DETAILS

**TITLE (PROVISIONAL)**	Appraising the causal effect of plasma caffeine on adiposity, type 2 diabetes and cardiovascular disease through Mendelian randomization
**AUTHORS**	Larsson, Susanna; Woolf, Benjamin; Gill, Dipender

VERSION 1 - REVIEW

**REVIEWER 1**	Martin, Susan. Competing Interest: None
**REVIEW RETURNED**	14-Sep-2022

**GENERAL COMMENTS**	This is a detailed MR study where the authors have looked at the causal effect of plasma caffeine levels on measures of adiposity and cardiovascular diseases. They only used two genetic variants as instruments, but used all the possible MR sensitivity analysis available to them in this situation. They explain clearly their choice of use of plasma caffeine levels compared to caffeine intake, and their use of variants within known genes compared to all significant genes. I have some minor points to be taken into consideration:<br><br>- “Individuals that carry genetic variants that lead to slower caffeine metabolism” – ‘associated with’ is better choice of words than ‘lead to’.<br>- “consume, on average, less coffee but have higher plasma caffeine levels.” – as you don’t explain your choice of plasma caffeine vs coffee intake until later on, this statement and the use of plasma caffeine may confuse the reader at this point – would recommend that some explanation of choice of plasma caffeine vs coffee intake is given earlier.<br>- “(MR) framework to investigate the effects” – change to ‘causal effects’.<br>- Maybe briefly explain the purpose of an MR for readers.<br>- Can you provide the FinnGen phenotype codes (and their associated ICD codes) for the traits you took from FinnGen – this information could be added to Table 1. Similarly, could you find and provide the ICD codes used for trait definitions for the other consortia given in Table 1?<br>- “dividing the SNP-outcome association estimate (beta coefficient or log odds ratio [OR]) with” – use ‘by’ rather than ‘with’.<br>- Why was fixed-effects meta-analysis used rather than random-effects? Random-effects should be the standard, with fixed-effects being used only if there is evidence of homogeneity. You do state that there was no heterogeneity between the studies – so maybe just add a few words to say this was behind your choice of using fixed-effects meta-analysis.<br>- “We estimated the effect of BMI on type 2 diabetes by selecting genome-wide significant SNPs in the GIANT BMI GWAS used above for the variant-exposure associations, and a fixed effect meta-analysis of FinnGen and DIAMANTE for the variant-outcome

	associations." – this sentence doesn't make sense, please revise.<br>- "genome-wide significant SNPs in the GIANT BMI GWAS" – give the number of SNPs here.<br>- "three 'pleiotropy robust' estimators" – describe this as a sensitivity analysis.<br>- "only two instrumental variables." – state this specifically as a limitation of study in the limitations section.<br>- "pleiotropic" – explain this term briefly to the audience.<br>- "phenome-wide association analysis" – how many phenotypes were used in total?<br>- "(Q<0.48, P>0.49 (Figure 2)." – missing end bracket.<br>- "no strong evidence" – change to 'no evidence'?<br>- You limited to "SNPs located in genes encoding enzymes with an established role in caffeine metabolism" – wouldn't a larger sample of SNPs be better, and then adjust for pleiotropy? Explain your decision here further, or maybe run secondary analysis.<br>- "MR analyses investigated cardiovascular outcomes" – change to 'investigating'.<br>- "our MR analyses investigated cardiovascular outcomes may have been under-powered." – briefly expand on why it was under-powered.<br>- "higher caffein levels" – spelling.<br>- "possible non-linear relations" – are there any methods you could use to adjust for this?<br>- Can you include the SEs for the SNP-caffeine associations in Sup Table 1?<br>- MR of BMI on T2D has been done many times before – maybe compare your results to some of these previous studies.<br>- Could you expand further in the Discussion about the meaning of your PheWAS results – specifically the metabolic biomarkers triglycerides, SHBG, ALT, etc.?<br>- Recommend that Table 1 be changed to Supplementary Table 1 – study characteristics better placed in supplemental material. And set the tables containing the main MR results as main Table 1, etc.

**REVIEWER 2**	Cornelis, Marilyn. Competing Interest: None
**REVIEW RETURNED**	02-Oct-2022

**GENERAL COMMENTS**	Dr. Larsson et al perform an MR of caffeine exposure on metabolic and cardiovascular traits. They use two variants near CYP1A2 and AHR which predict higher circulating caffeine levels and lower dietary caffeine consumption behavior. These variants have already been used as caffeine exposure/behavior IVs for dozens of traits and diseases, including those of interest in the current study. The methods employed are standard to the field. Although the sample size in the current work is larger than some prior publications the study lacks novelty and the knowledge gained is minimal. Taking the high impact of BMJ Medicine into consideration, I think this paper is better suited for a different journal.

**REVIEWER 3**	Caro, Ilana; University of Bordeaux, Bordeaux Population Health reasearch center. Competing Interest: None
**REVIEW RETURNED**	20-Oct-2022

**GENERAL COMMENTS**	This article discusses an interesting and relevant topic. Caffeine being a widely used psychoactive substance, studying its effects in

	a comprehensive way is essential and needed to decipher its action on multiple diseases. Coffee consumptions and caffeine levels have been studied in observational studies and randomized controls trials (RCTs) and have been implicated in weight-related outcomes such as body mass index, weight, fat mass reduction but also reduction of adiposity related disorders such as type 2 diabetes and cardiovascular disease. Those studies however, couldn't consider confounders nor infer causality, making the present study highly relevant.<br><br>Regarding the methods used in this manuscript, mendelian randomization is a well-known technique. However, the follow-up analysis and methods you are using aren't that known to the reader. Therefore, deeper description of those methods is needed especially for the two-step network MR mediation analysis and the phenome-wide association analysis. Some specific suggests are listed in suggested revisions at the end of this review.<br><br>The results are clear and well described and the discussion is well-constructed.<br><br>Comments:<br><br><u>Abstract</u><br><br>- More thorough descriptions of the participants need to be included in the manuscript (specify the cohorts used, the phenotype observed in, and the sample size of those cohorts)<br>- More thorough descriptions of the main outcomes need to be included in the manuscript (specify the sample size for each outcome, specify that you are using summary statistics of genome-wide association studies)<br><br><u>Introduction</u><br><br>- At the beginning of the introduction, it would be interesting to expand the sentence "considering the extensive intake of coffee" with some reference<br><br><u>Statistical analysis</u><br><br>- I suggest you add a formula in the first section to make it clearer for the reader in the first part of this section (l.12-22)<br>- Same for section: l.43-45<br>- Why did the authors not perform colocalization analyses.<br><br><u>Results</u><br><br>- You used the weighted median estimator for the mediation analysis but you said in the statistical analysis that three pleiotropy robust estimators: MR Egger, weighted median and weighted mode were used. I suggest that even if you use only results of the weighted median for the result part, you present the results of the 3 methods in a table.<br>- In your paper you talk about the "long-term effect" of increased plasma caffeine: can you elaborate on why this work allows the study of **<u>long-term</u>** effect?<br><br><u>Discussion</u>

	- *Principal findings*: Last sentence of the first part (l.5-7): "There was no strong evidence of an association with ischemic heart disease, atrial fibrillation, heart failure, and stroke.", I suggest to add "**of genetically predicted higher plasma caffeine level**" to be more specific.<br><br>- *Comparison with other studies*:<br>o (l.29, p.9) "been examined in many observational studies", can you add the studies you are referring to?<br>o In the part (l. 56-6, p.9-10), about the genetic variants being associated with higher plasma caffeine levels also associated with lower coffee and caffeine consumptions, I suggest emphasizing this part in your introduction when describing and choosing instruments as you do here in the discussion.<br>o (l.19-24, p.10) need of a reference for those "studies based on a larger set of instrumental variables including several SNPs in genes with a less clear or unknown role in predicting higher coffee and caffeine consumption"<br><br>- *Biological processes*<br>o For this part, it would be more appropriate to assess the discussion with a graphic representation of the potential biological processes implicated. It will be clearer for the reader but also for the conclusions you are making.<br><br>- *Strengths and limitations of this study*<br>o For the first sentence (l. 44-47, p.11): could you elaborate on the methods you employed to diminish bias due to confounding and reverse causation?<br><br>Figures<br><br>- Supplementary figure 2: Could you state the numeric values of the Cochrane Q pvalues instead of "<0.00.1"?

**REVIEWER 4**	Riley, Richard; University of Birmingham, Institute of Applied Health Research. Competing Interest: None
**REVIEW RETURNED**	04-Nov-2022

**GENERAL COMMENTS**	Thank you for the opportunity to review this interesting paper, on a very important topic. I am not a causal inference expert, and so the paper should be reviewed by a methodologist with such expertise, but I have the following comments for the authors to consider moving forward:<br><br>1) Fat-mass and fat-free mass are strongly related to each other, so I do not understand how the conclusion can be that "Higher genetically-predicted plasma caffeine levels were associated with lower body mass index and whole-body fat mass (P<0.001) but not fat-free mass" – surely if it is associated with one, it is associated with the other?<br><br>2) The results section jumps straight into the results, but we need details of the studies, their characteristics, the case-mix, and so forth to get an understanding of the context here – otherwise it is very black-box.<br><br>3) The authors are using SNP-outcome association estimates

	obtained from 6 previous studies – but we need details of the estimates in each of those studies too, and the presentation of them on a forest plot with the pooled estimate for each outcome. Same with the SNP-caffeine estimate. We need clarity on the data being fed into the modelling. The forest plots currently shown are based on pooling the two estimates post meta-analysis of the 6 studies, so we are missing the information behind this.<br><br>4) Fixed-effects should be fixed-effect meta-analysis (as the authors are assuming one fixed effect).<br><br>5) The authors claim there is no evidence of heterogeneity, with p = 0.12 based in the Q test. But this is based on only 2 studies, so will always have low power – I do think a sensitivity analysis allowing for heterogeneity via a random-effects model may be useful too, therefore. When there is detected heterogeneity, it still was not clear if this was being accounted for when using the 'weighted median estimator'.<br><br>6) Are the 6 studies providing evidence good quality? If this was a systematic review, we would expect a formal risk of bias assessment of each study, to know if the study methodology was deemed appropriate. Hence, this is an important gap that needs to be addressed in any revision. How can we know that confounding was appropriately addressed in the studies? I assume not every MR study is equally reliable. How was missing data handled, different lengths of follow-up, and so forth?<br><br>7) Is Figure 3 essentially a DAG? I think a DAG is needed upfront, to put this in a causal inference context more clearly.<br><br>I hope these comments are helpful for the authors and BMJ Medicine moving forwards.

**REVIEWER 5**	Davies, Neil; University of Bristol. Competing Interest: I know and have co-authored papers with Dipender Gill, and know Benjamin Woolf, who worked at the same institute as me. I informed the editor of this CoI before reviewing it.
**REVIEW RETURNED**	10-Nov-2022

**GENERAL COMMENTS**	This is a high quality Mendelian randomization study. The findings are novel and interesting primarily because they're using plasma caffeine levels rather than questionnaire measures of coffee consumption. This overcomes the problem that individuals who consume the same amount of coffee may have very different levels of caffeine in the blood.<br><br>I have three, hopefully useful points:<br><br>1) The overall results look credible, but the paper would benefit from following the STROBE-MR guidelines, and provide some assessment of the likely quality of the GWAS used in the paper see here:<br><br>https://jamanetwork.com/journals/jama/article-abstract/2785494<br><br>2) In the discussion, the authors could provide a more nuanced discussion of the statistical power of their cardiovascular results. It's

	not a statistical power issue here, the results are actually reasonably precise and I would imagine if you tested for it you'd find consistent evidence that the estimated effects of plasma caffeine levels on diabetes are greater than for the cardiovascular disease outcomes. You could also interpret the confidence interval of the effect estimates for cardiovascular disease as the largest protective/harmful effects consistent with the estimate given 80% power etc. For IHD and AF these results suggest caffeine plasma levels are unlikely to be larger than 15% and 12%, or more harmful than 1% and 5% increases respectively. This would just require a minor textual change.<br><br>3) How can you exclude the possibility that your results are due to people with these variants drinking less coffee? I.e. it has nothing to do with the caffeine, but some other compound in coffee which increases risk of diabetes? The variants have at least two effects, 1) people with the variants have higher levels of blood caffeine, and 2) drink less coffee. How can we know which hypothesis is correct. Note, I'm not sure that there's much in the way of analysis you could do on this point (e.g. MVMR with cups of coffee per day + plasma caffeine is likely to be woefully underpowered) so perhaps it's just a case of noting this as a potential limitation/explanation for your results.<br><br>I hope these comments help improve the paper.

VERSION 1 – AUTHOR RESPONSE

Reviewer: 1

This is a detailed MR study where the authors have looked at the causal effect of plasma caffeine levels on measures of adiposity and cardiovascular diseases. They only used two genetic variants as instruments, but used all the possible MR sensitivity analysis available to them in this situation. They explain clearly their choice of use of plasma caffeine levels compared to caffeine intake, and their use of variants within known genes compared to all significant genes. I have some minor points to be taken into consideration:

Response: Thank you for reviewing our manuscript and for the constructive comments and suggestions that helped us to improve the paper.

- “Individuals that carry genetic variants that lead to slower caffeine metabolism” – ‘associated with’ is better choice of words than ‘lead to’.

Response: We have changed to “associated with” in this sentence.

- “consume, on average, less coffee but have higher plasma caffeine levels.” – as you don’t explain your choice of plasma caffeine vs coffee intake until later on, this statement and the use of plasma caffeine may confuse the reader at this point – would recommend that some explanation of choice of plasma caffeine vs coffee intake is given earlier.

Response: We agree and have expanded this statement already in the Introduction after that sentence. The extended text in the Introduction is as following:

"Individuals that carry genetic variants that associate with slower caffeine metabolism consume, on average, less coffee but have higher plasma caffeine levels.[20] This phenomenon likely relates to individuals with a slow metabolism of caffeine consequently consuming less coffee/caffeine than those with a fast caffeine metabolism to achieve or retain the levels of caffeine required for desired psychostimulant effects.[20]"

- *"(MR) framework to investigate the effects" – change to 'causal effects'.*

Response: We have changed to "causal effects" as suggested.

- Maybe briefly explain the purpose of an MR for readers.

Response: We have now briefly explained the purpose of an MR study. The following text has been added to the last paragraph of the Introduction section:

"The purpose of an MR analysis is to improve causal inference by utilizing genetic variants that are reliably and strongly associated with the exposure as unbiased proxy indicators. As genetic variants are fixed at conception, individuals with genetic variants that associate with higher plasma caffeine will, on average, be exposed to higher caffeine levels throughout life compared with those with the variants associated with lower plasma caffeine."

- Can you provide the FinnGen phenotype codes (and their associated ICD codes) for the traits you took from FinnGen – this information could be added to Table 1. Similarly, could you find and provide the ICD codes used for trait definitions for the other consortia given in Table 1?

Response: As recommended, we have now provided the FinnGen phenotype codes as well as the ICD codes used for disease definitions in supplemental table 1.

- "dividing the SNP-outcome association estimate (beta coefficient or log odds ratio [OR]) with" – use 'by' rather than 'with'.

Response: We have changed to "by" in this sentence.

- Why was fixed-effects meta-analysis used rather than random-effects? Random-effects should be the standard, with fixed-effects being used only if there is evidence of homogeneity. You do state that there was no heterogeneity between the studies – so maybe just add a few words to say this was behind your choice of using fixed-effects meta-analysis.

Response: We have now used random-effects model instead of the fixed-effect model for the meta-analysis. The 95% confidence interval for type 2 diabetes only slightly widened, from “0.77 to 0.86” to “0.74 to 0.89”. The confidence interval for the cardiovascular disease outcomes remained identical. We have now updated the text and Figure 2.

- “We estimated the effect of BMI on type 2 diabetes by selecting genome-wide significant SNPs in the GIANT BMI GWAS used above for the variant-exposure associations, and a fixed effect meta-analysis of FinnGen and DIAMANTE for the variant-outcome associations.” – this sentence doesn’t make sense, please revise.

Response: We have now revised this part to read:

“When estimating the association of genetically predicted BMI with type 2 diabetes, we selected as instrumental variables genome-wide significant SNPs from the GIANT BMI GWAS, as described above. For the type 2 diabetes genetic association estimates, we performed a random-effects meta-analysis of FinnGen and DIAMANTE.”

- “genome-wide significant SNPs in the GIANT BMI GWAS” – give the number of SNPs here.

Response: We have now added in the manuscript that 483 SNPs were used for BMI.

- “three ‘pleiotropy robust’ estimators” – describe this as a sensitivity analysis.

Response: We have now clarified that the “three ‘pleiotropy robust’ estimators” were a sensitivity analysis.

- “only two instrumental variables.” – state this specifically as a limitation of study in the limitations section.

Response: We have now mentioned in the limitation section that a shortcoming is the use of only two SNPs for caffeine exposure which reduced the power of the analyses.

- “pleiotropic” – explain this term briefly to the audience.

Response: We have amended this sentence and briefly explained pleiotropy. The revised sentence is:

“To assess potential pleiotropy (i.e., when a genetic variant associates with more than one phenotype)…”

- “phenome-wide association analysis” – how many phenotypes were used in total?

Response: The number of phenotypes identified for the two plasma caffeine-associated SNPs has been included the Results section:

“In the phenome-wide association analysis performed in the MR-Base platform, 39 763 and 40 553 phenotype associations were available for the CYP19A1 and the AHR genetic variant, respectively.”

- “(Q<0.48, P>0.49 (Figure 2).” – missing end bracket.

Response: The end bracket has been added.

- “no strong evidence” – change to ‘no evidence’?

Response: We have changed to “no evidence”.

- You limited to “SNPs located in genes encoding enzymes with an established role in caffeine metabolism” – wouldn’t a larger sample of SNPs be better, and then adjust for pleiotropy? Explain your decision here further, or maybe run secondary analysis.

Response: In our opinion, using biologically plausible variants that have an established role in caffeine metabolism is preferred as this reduces risk of incorporating pleiotropic variants and conforms to the MR assumptions. Using a wider selection of potentially invalid instruments would be unlikely to improve the study, particularly if the majority of variants are invalid and InSIDE is violated (which is usually the case).

- “MR analyses investigated cardiovascular outcomes” – change to ‘investigating’.

Response: We have changed to “investigating” in this sentence.

- “our MR analyses investigated cardiovascular outcomes may have been under-powered.” – briefly expand on why it was under-powered.

Response: Thank you for this comment. We have now provided a more nuanced discussion of the statistical power of the cardiovascular outcomes results as following:

"The 95% CI for the cardiovascular outcomes suggested that any possible protective effect of plasma caffeine levels on ischemic heart disease and atrial fibrillation are unlikely to be larger than 15% and 12%, or more harmful than 1% and 5% increases, respectively."

- "higher caffein levels" – spelling.

Response: We have corrected to "caffeine".

- "possible non-linear relations" – are there any methods you could use to adjust for this?

Response: Investigations of non-linear relations would require individual level participant data for both caffeine levels and the outcomes, which we do not currently have access to and unfortunately are unable to obtain.

- Can you include the SEs for the SNP-caffeine associations in Sup Table 1?

Response: SEs were not provided in the caffeine GWAS and insufficient data were available to calculate them. The SEs for the SNP-caffeine associations are not needed in MR analysis (only the SEs for the SNP-outcome associations are necessary). As SNPs used in the MR study were associated with caffeine levels at genome-wide significance, the variance in the estimated results is primarily driven by the variance in the effect estimates for the outcome rather than the exposure.

- MR of BMI on T2D has been done many times before – maybe compare your results to some of these previous studies.

Response: As suggested, we have now compared our MR results for BMI in relation to T2D with those from previous MR studies. The following text has been added in the Discussion section:

"As in previous MR studies,[33] we found a strong association between genetically predicted body mass index and type 2 diabetes risk. The magnitude of the association was somewhat stronger in our MR analysis than in a previous MR studies,[33] which is likely related to different instruments used for body mass index and different data source for type 2 diabetes."

- Could you expand further in the Discussion about the meaning of your PheWAS results – specifically the metabolic biomarkers triglycerides, SHBG, ALT, etc.?

Response: As suggested, we have discussed those biomarkers in the Discussion section. The following text was added:

"The genetic alleles that associated with higher plasma caffeine levels associated with lower coffee and tea consumption. Thus, an alternative mechanism is that individuals who carry those alleles have a lower risk of type 2 diabetes, compared with those with the other sets of alleles, due to lower exposure to any other substances in coffee (e.g., diterpenes) or tea that increase the risk of type 2 diabetes. With respect to this, the plasma caffeine-raising allele for the SNP in the *AHR* locus was associated with lower levels of triglycerides, low-density lipoprotein cholesterol, and sex-hormone binding globulin adjusted for BMI as well as higher levels of bilirubin and alkaline phosphatase. The association with triglycerides and cholesterol might be related to lipid-raising diterpenes (i.e., cafestol and kahweol) present in unfiltered coffee.[48] Coffee and caffeine consumption has been reported to associate with sex-hormone binding globulin[49] as well as certain biomarkers of liver function and liver cancer.[50]"

- Recommend that Table 1 be changed to Supplementary Table 1 – study characteristics better placed in supplemental material. And set the tables containing the main MR results as main Table 1, etc.

Response: Thank you for this suggestion. We agree and have made the corresponding changes.

Reviewer: 2

Dr. Larsson et al perform an MR of caffeine exposure on metabolic and cardiovascular traits. They use two variants near CYP1A2 and AHR which predict higher circulating caffeine levels and lower dietary caffeine consumption behavior. These variants have already been used as caffeine exposure/behavior IVs for dozens of traits and diseases, including those of interest in the current study. The methods employed are standard to the field. Although the sample size in the current work is larger than some prior publications the study lacks novelty and the knowledge gained is minimal. Taking the high impact of BMJ Medicine into consideration, I think this paper is better suited for a different journal.

Response: Although there are several MR studies on coffee consumption and different diseases, those studies included smaller sample sizes (and could not exclude that low power explained the null findings) and few studies looked at blood caffeine levels specifically. Moreover, this is to the best of our knowledge the first MR study revealing the significant causal effect of higher caffeine levels on lower body mass index and fat mass and reduced risk of type 2 diabetes. In addition, a novel finding is that we observe that the association between caffeine and type 2 diabetes is to some extent (estimated 43%) mediated by body mass index. As the mechanisms underpinning the observational association of coffee/caffeine intake with type 2 diabetes have not been clearly defined, this mediating effect is of notable importance. We believe that our comprehensive MR study and PheWAS, which show novel findings, are of great public and clinical interest and would also be of interest to the readership of BMJ Medicine.

Reviewer: 3

This article discusses an interesting and relevant topic. Caffeine being a widely used psychoactive substance, studying its effects in a comprehensive way is essential and needed to decipher its action on multiple diseases. Coffee consumptions and caffeine levels have been studied in observational studies and randomized controls trials (RCTs) and have been implicated in weight-related outcomes such as body mass index, weight, fat mass reduction but also reduction of adiposity related disorders such as type 2 diabetes and cardiovascular disease. Those studies however, couldn't consider confounders nor infer causality, making the present study highly relevant.

Regarding the methods used in this manuscript, mendelian randomization is a well-known technique. However, the follow-up analysis and methods you are using aren't that known to the reader. Therefore, deeper description of those methods is needed especially for the two-step network MR mediation analysis and the phenome-wide association analysis. Some specific suggests are listed in suggested revisions at the end of this review.

The results are clear and well described and the discussion is well-constructed.

Response: Thank you for reviewing our paper and for helpful comments and suggestions that helped us to improve the work. Please see below our responses and changes made following each specific comment.

Abstract
- More thorough descriptions of the participants need to be included in the manuscript (specify the cohorts used, the phenotype observed in, and the sample size of those cohorts)
- More thorough descriptions of the main outcomes need to be included in the manuscript (specify the sample size for each outcome, specify that you are using summary statistics of genome-wide association studies)

Response: We have now provided detailed information for the GWAS consortia and studies, including the phenotypes (e.g., ICD codes), sample size etc., used for the MR analysis in supplemental table 1. We have also clarified that we used summary statistics data.

Introduction
- At the beginning of the introduction, it would be interesting to expand the sentence "considering the extensive intake of coffee" with some reference.

Response: We have now added three references here.

Statistical analysis
- I suggest you add a formula in the first section to make it clearer for the reader in the first part of this section (l.12-22)
- Same for section: l.43-45
- Why did the authors not perform colocalization analyses.

Response: We have cited the original papers that give the formula as well as the packages used to run the analyses. Adding formulas in the text could detract from more relevant information.

Colocalization was not suitable in this study as there are two variants in different genomic regions, and colocalization is typically only applicable to a single gene region. Moreover, as it requires both variants together to give sufficient statistical power for Mendelian randomization analyses, considering each variant alone will likely offer insufficient statistical power to make colocalization analysis informative or helpful.

Results

- You used the weighted median estimator for the mediation analysis but you said in the statistical analysis that three pleiotropy robust estimators: MR Egger, weighted median and weighted mode were used. I suggest that even if you use only results of the weighted median for the result part, you present the results of the 3 methods in a table.
- In your paper you talk about the "long-term effect" of increased plasma caffeine: can you elaborate on why this work allows the study of long-term effect?

Response: We have provided the results for all three pleiotropy robust estimators (MR Egger, weighted median and weighted mode) in supplemental table 3.

We have now clarified that genetic variants are fixed at birth and that the results of an MR analysis therefore provide the long-term (life-long) effect of the exposure on the outcome. Individuals with the genetic variants associated with higher plasma caffeine levels will, on average, have higher plasma caffeine levels compared with those with the other sets of variants throughout life. The following sentence was added in the end of the Introduction:

"As genetic variants are fixed at conception, individuals with genetic variants that associate with higher plasma caffeine will, on average, be exposed to higher caffeine levels throughout life compared with those with the variants associated with lower plasma caffeine."

Discussion

Principal findings

Last sentence of the first part (l.5-7): "There was no strong evidence of an association with ischemic heart disease, atrial fibrillation, heart failure, and stroke.", I suggest to add "of genetically predicted higher plasma caffeine level" to be more specific.

Response: We have amended the sentence as suggested.

Comparison with other studies

o (l.29, p.9) "been examined in many observational studies", can you add the studies you are referring to?

Response: We have added the reference to one of the most recent comprehensive meta-analysis of observational studies of coffee consumption and type 2 diabetes risk.

o In the part (l. 56-6, p.9-10), about the genetic variants being associated with higher plasma caffeine levels also associated with lower coffee and caffeine consumptions, I suggest emphasizing this part in your introduction when describing and choosing instruments as you do here in the discussion.

Response: We have clarified in the Introduction section that individuals that carry genetic variants that associate with slower caffeine metabolism consume, on average, less coffee but have higher plasma caffeine levels.

o (l.19-24, p.10) need of a reference for those "studies based on a larger set of instrumental variables including several SNPs in genes with a less clear or unknown role in predicting higher coffee and caffeine consumption"

Response: We have now added the references that we refer to in this sentence.

- Biological processes

o For this part, it would be more appropriate to assess the discussion with a graphic representation of the potential biological processes implicated. It will be clearer for the reader but also for the conclusions you are making.

Response: While we appreciate the reviewers point about facilitating communication of the findings, we would prefer to refrain from this figure at present. The reason for this being that this is genetic investigation that makes several assumptions as discussed in the manuscript. While the objective is indeed towards inferring causal effects, the possible underlying mechanisms and biological pathways were not studied in this study.

- Strengths and limitations of this study

o For the first sentence (l. 44-47, p.11): could you elaborate on the methods you employed to diminish bias due to confounding and reverse causation?

Response: As suggested, we have now clarified why the MR design diminishes bias due to reverse causation and confounding. The revised text is as follows:

"An important strength of this study is the MR design, which minimizes bias due to reverse causation (as genetic variants are fixed at conception and cannot be changed by disease status) and reduces potential effects of confounding factors (as the genetic variants associated with the exposure under study are generally not associated with environmental exposures and other self-adopted behaviors, except for caffeine-containing beverage consumption in this MR study)."

Figures

- Supplementary figure 2: Could you state the numeric values of the Cochrane Q pvalues instead of "<0.001"?

Response: Added as suggested.

Reviewer: 4

Thank you for the opportunity to review this interesting paper, on a very important topic. I am not a causal inference expert, and so the paper should be reviewed by a methodologist with such expertise, but I have the following comments for the authors to consider moving forward:

Response: We thank the Reviewer for their time and constructive comments.

1) Fat-mass and fat-free mass are strongly related to each other, so I do not understand how the conclusion can be that "Higher genetically-predicted plasma caffeine levels were associated with lower body mass index and whole-body fat mass (P<0.001) but not fat-free mass" – surely if it is associated with one, it is associated with the other?

Response: We have now modified the text to write:

"Genetically-predicted higher plasma levels of caffeine were associated with lower BMI (beta -0.08 SDs [1 SD equals ~4.8 kg/m2] for every SD increase in plasma caffeine; 95% confidence interval [CI] -0.10 to -0.06; P<0.001) and whole-body fat mass (beta -0.06 SDs [1 SD equals ~9.5 kg] for every SD increase in plasma caffeine; 95% CI -0.08 to -0.04; P<0.001) but had weaker and non-significant association with fat-free mass (beta -0.01 SDs [1 SD equals ~11.5 kg] for every SD increase in plasma caffeine; 95% CI -0.02 to -0.00; P=0.17)"

2) The results section jumps straight into the results, but we need details of the studies, their characteristics, the case-mix, and so forth to get an understanding of the context here – otherwise it is very black-box.

Response: We have now provided the important details for the consortia and studies used for the MR analyses in Supplemental table 1.

3) The authors are using SNP-outcome association estimates obtained from 6 previous studies – but we need details of the estimates in each of those studies too, and the presentation of them on a forest plot with the pooled estimate for each outcome. Same with the SNP-caffeine estimate. We need clarity on the data being fed into the modelling. The forest plots currently shown are based on pooling the two estimates post meta-analysis of the 6 studies, so we are missing the information behind this.

Response: We have provided the estimates from all consortia and studies in Supplemental Table 2, and presented the MR estimate for each of them as well as the pooled estimates in a forest plot (Figure 2).

4) Fixed-effects should be fixed-effect meta-analysis (as the authors are assuming one fixed effect).

Response: We have changed to a random-effects model, as suggested in the comment below.

5) The authors claim there is no evidence of heterogeneity, with p = 0.12 based in the Q test. But this is based on only 2 studies, so will always have low power – I do think a sensitivity analysis allowing for heterogeneity via a random-effects model may be useful too, therefore. When there is detected heterogeneity, it still was not clear if this was being accounted for when using the 'weighted median estimator'.

Response: We have now used random-effects model instead of the fixed-effect model for the meta-analysis. The 95% confidence interval for type 2 diabetes only slightly widened, from "0.77 to 0.86" to "0.74 to 0.89". The confidence interval for the cardiovascular disease outcomes remained identical. We have now updated the text and Figure 2.

6) Are the 6 studies providing evidence good quality? If this was a systematic review, we would expect a formal risk of bias assessment of each study, to know if the study methodology was deemed appropriate. Hence, this is an important gap that needs to be addressed in any revision. How can we know that confounding was appropriately addressed in the studies? I assume not every MR study is equally reliable. How was missing data handled, different lengths of follow-up, and so forth?

Response: We have now summarized the most important information, such as population (ancestry, sample size, etc.), ICD codes for the outcomes, and adjustments for each study in supplemental table 1. Some information that may be important for observational studies are of less importance for MR studies, such as adjustments (as an MR analysis in theory are free of confounding as genetic variants associated with one phenotype are generally unrelated to environmental factors etc.) and follow-up time (as the genetic exposure is already present at birth, any disease that occurs after birth is an "incident" case). Adjustments in a GWAS should be kept to a minimum number of factors, such as age, sex, and genetic principal components, to avoid collider bias in future MR analyses. The reliability of an MR analysis is mostly dependent on the genetic variants used (they should be strongly associated with exposure and ideally located in one or more relevant gene region, as in the present MR analysis) and the sample size of the included studies to have sufficient power in the analysis (as in the present MR analysis based on large sample sizes, as shown in supplemental table 1).

7) Is Figure 3 essentially a DAG? I think a DAG is needed upfront, to put this in a causal inference context more clearly.

Response: We have now clarified in the title of figure 3 that this is a causal directed acyclic graph (DAG) showing the total effect of plasma caffeine on type 2 diabetes risk as well as the effect mediated by body mass index.

I hope these comments are helpful for the authors and BMJ Medicine moving forwards.
Best wishes, Prof Richard Riley
Chief Statistics Editor, BMJ Medicine

Response: We are very grateful for these helpful comments that improved our paper.

Reviewer: 5

This is a high quality Mendelian randomization study. The findings are novel and interesting primarily because they're using plasma caffeine levels rather than questionnaire measures of coffee consumption. This overcomes the problem that individuals who consume the same amount of coffee may have very different levels of caffeine in the blood.

I have three, hopefully useful points:

1) The overall results look credible, but the paper would benefit from following the STROBE-MR guidelines, and provide some assessment of the likely quality of the GWAS used in the paper see here:

https://eur01.safelinks.protection.outlook.com/?url=https%3A%2F%2Fjamanetwork.com%2Fjournals%2Fjama%2Farticle-abstract%2F2785494&data=05%7C01%7Csusanna.larsson%40ki.se%7Ca444032476ed45b404a908dac880931f%7Cbff7eef1cf4b4f32be3da1dda043c05d%7C0%7C0%7C638042752747900385%7CUnknown%7CTWFpbGZsb3d8eyJWIjoiMC4wLjAwMDAiLCJQIjoiV2luMzIiLCJBTiI6Ik1haWwiLCJXVCI6Mn0%3D%7C3000%7C%7C%7C&sdata=05H7JyfYmDl7K%2FkwnHWBrJizpO57BFdfuA3rDOI9RcU%3D&reserved=0

Response: As recommended we have now reported the results following the STROBE-MR guidelines. The checklist has also been added.

2) In the discussion, the authors could provide a more nuanced discussion of the statistical power of their cardiovascular results. It's not a statistical power issue here, the results are actually reasonably precise and I would imagine if you tested for it you'd find consistent evidence that the estimated effects of plasma caffeine levels on diabetes are greater than for the cardiovascular disease outcomes. You could also interpret the confidence interval of the effect estimates for cardiovascular disease as the largest protective/harmful effects consistent with the estimate given 80% power etc. For IHD and AF these results suggest caffeine plasma levels are unlikely to be larger than 15% and 12%, or more harmful than 1% and 5% increases respectively. This would just require a minor textual change.

Response: Thank you for this insightful point. We agree and now provide a more nuanced discussion of the statistical power of the cardiovascular results. The following sentence has been added:

"The 95% CI for the cardiovascular outcomes suggested that any possible protective effect of plasma caffeine levels on ischemic heart disease and atrial fibrillation are unlikely to be larger than 15% and 12%, or more harmful than 1% and 5% increases, respectively."

3) How can you exclude the possibility that your results are due to people with these variants drinking less coffee? I.e. it has nothing to do with the caffeine, but some other compound in coffee which increases risk of diabetes? The variants have at least two effects, 1) people with the variants have higher levels of blood caffeine, and 2) drink less coffee. How can we know which hypothesis is correct. Note, I'm not sure that there's much in the way of analysis you could do on this point (e.g. MVMR with cups of coffee per day + plasma caffeine is likely to be woefully underpowered) so perhaps it's just a case of noting this as a potential limitation/explanation for your results.

Response: We have now discussed this possibility in the Discussion section. The following text was added:

"The genetic alleles that associated with higher plasma caffeine levels associated with lower coffee and tea consumption. Thus, an alternative mechanism is that individuals who carry those alleles have a lower risk of type 2 diabetes, compared with those with the other sets of alleles, due to lower exposure to any other substances in coffee (e.g., diterpenes) or tea that increase the risk of type 2 diabetes."

I hope these comments help improve the paper.

Response: Thank you for reviewing our paper and for these very helpful comments that certainly improved our paper.

VERSION 2 – REVIEW

**REVIEWER 4**	Riley, Richard; University of Birmingham, Institute of Applied Health Research. Competing Interest: None
**REVIEW RETURNED**	09-Dec-2022

**GENERAL COMMENTS**	The revision and response to my comments are generally clear and excellent.<br><br>My only comment is in regards to heterogeneity. Firstly, what random effects modelling approach was used to combine the two studies? DerSimonian and Laird? Please explain.<br><br>Also – and this may be due to my lack of understanding about what is happening in the data sources and pooling - but given that the authors test for heterogeneity, and so are clearly interested in whether results vary across studies, why does it make sense to just look at the heterogeneity between the 2 studies (the 'FinnGen' one based on the previous evidence and the 1 from this study), and not to look at the 6 studies (from previous evidence/studies/cohorts,

	unpooled) and the 1 from this study? In other words, why pool the previous 6 studies, and then pool again with the new study, rather than pooling all 7 studies in a random effects meta-analysis? I think I must be getting confused about what is happening in the methods, but I just wanted to raise this to gain a better understanding about how and WHEN different sources of evidence are being combined, and why heterogeneity is investigated at the end, and not for the evidence toward the two studies being pooled. Perhaps we could add about the different cohorts and studies into Figure 1, to explain where they each come into play.

VERSION 2 – AUTHOR RESPONSE

Reviewer 4

The revision and response to my comments are generally clear and excellent.

My only comment is in regards to heterogeneity. Firstly, what random effects modelling approach was used to combine the two studies? DerSimonian and Laird? Please explain.

Response: Yes, the DerSimonian and Laird method was used. We have now clarified this in the manuscript (page 7, second line).

Also – and this may be due to my lack of understanding about what is happening in the data sources and pooling - but given that the authors test for heterogeneity, and so are clearly interested in whether results vary across studies, why does it make sense to just look at the heterogeneity between the 2 studies (the 'FinnGen' one based on the previous evidence and the 1 from this study), and not to look at the 6 studies (from previous evidence/studies/cohorts, unpooled) and the 1 from this study? In other words, why pool the previous 6 studies, and then pool again with the new study, rather than pooling all 7 studies in a random effects meta-analysis? I think I must be getting confused about what is happening in the methods, but I just wanted to raise this to gain a better understanding about how and WHEN different sources of evidence are being combined, and why heterogeneity is investigated at the end, and not for the evidence toward the two studies being pooled. Perhaps we could add about the different cohorts and studies into Figure 1, to explain where they each come into play.

Response: We thank the reviewer for raising this and offering us the opportunity to explain. Is not appropriate to pool our MR results with those from previous conventional observational studies, because they are measuring different estimates (i.e., association of genetically predicted blood caffeine levels in our present MR study vs. self-reported caffeine intake estimated from coffee/tea consumption in previous studies). As our current work is an MR study, we only pool the MR estimates.
